# Clear cell sarcoma of the jejunum: a case report

**DOI:** 10.1186/1477-7819-11-17

**Published:** 2013-01-25

**Authors:** Konstantinos Lasithiotakis, Alexandros Protonotarios, Vasiliki Lazarou, Maria Tzardi, George Chalkiadakis

**Affiliations:** 1 Department of General Surgery, University Hospital of Heraklion, Medical School of Heraklion, University of Crete, Heraklion, Greece; 2 Department of Pathology, University Hospital of Heraklion, Medical School of Heraklion, University of Crete, Heraklion, Greece

**Keywords:** Clear cell sarcoma, Jejunum

## Abstract

**Background:**

Clear cell sarcoma (CCS), also known as malignant melanoma of soft parts, is a rare type of soft tissue sarcoma which exhibits morphological, immunohistochemical and ultrastructural similarity with malignant melanoma. It is rarely localized in the intestine and the natural history of this tumor is not yet clear.

**Case report:**

A 49-year-old woman presented with diffuse abdominal colicky pain and vomitus over the previous seven days. An X-ray of the abdomen revealed obstruction of the small intestine. The patient underwent contrast enhanced abdominal computerized tomography (CT), which confirmed the obstruction at the jejunum and an associated circumferential wall thickening extending about 3 cm in length, causing concentric narrowing of the lumen. At laparotomy, a mass was recognized at the level of the jejunum in the small intestine, which caused almost complete obstruction of the lumen. At the point of obstruction, adhered loops of small intestine were found. A segmental small bowel resection was performed with 5 cm clear margins and its respective mesenteric lymph nodes.

**Results:**

Histological examination of the specimen revealed a tumor (3×3×2cm) with epithelioid cell characteristics and eosinophilic or clear cytoplasm and focal translucent nuclei. Immunohistochemistry was positive for S100, epithelial membrane antigen (EMA) and synaptophysin. The tumor was pankeratin AE1/AE2, GFAP, HMB45 and MART-1/Melan-A negative. Twelve lymph nodes were retrieved and were free of neoplastic infiltration. Cytogenetic examination revealed translocation of the *EWSR1* gene. The patient had an uncomplicated postoperative course and left the hospital seven days after her admission in good general condition. After 20 months of follow-up the patient remains asymptomatic without any clinical or radiological evidence of recurrence.

**Conclusion:**

CCS sarcoma can be rarely localized in the jejunum. Due to its morphological similarity to malignant melanoma, cytogenetic examination is necessary for its diagnosis. Wide resection of the tumor and its respective lymph nodes was associated with a 20-month disease free survival in this patient.

## Background

Clear cell sarcoma (CCS) is a rare type of soft tissue sarcoma, originally described by Enzinger in 1965 [1]. The main characteristic of CCS is its morphological, immunohistochemical and ultrastructural similarity to malignant melanoma [2]. However, CCS is genetically distinct from melanomas as it lacks *BRAF* mutations [3] and in the majority of cases it harbors a chromosomal translocation t(12;22)(q13;q12), which leads to the formation of the EWS/ATF1 fusion transcript [4]. The tumor typically arises in the distal extremities of young adults involving, almost always, tendons and aponeuroses. Gastrointestinal (GI) tract involvement, as the primary site has rarely been reported in the literature [5]. Thus, the natural history and the exact prognosis of these tumors are not yet clear. Herein we present a case of CCS of the jejunum and its 20-month follow-up.

## Case presentation

A 49-year-old female patient presented to the emergency department with a seven-day history of diffuse abdominal colicky pain and continuous vomiting. On clinical examination, the abdomen was distended, exhibiting metallic bowel sounds. An X-ray was performed which indicated obstruction of the small intestine. Computerized tomography showed obstruction at the distal part of the jejunum and eccentric wall thickening, measuring 3 cm.

**Figure 1:**
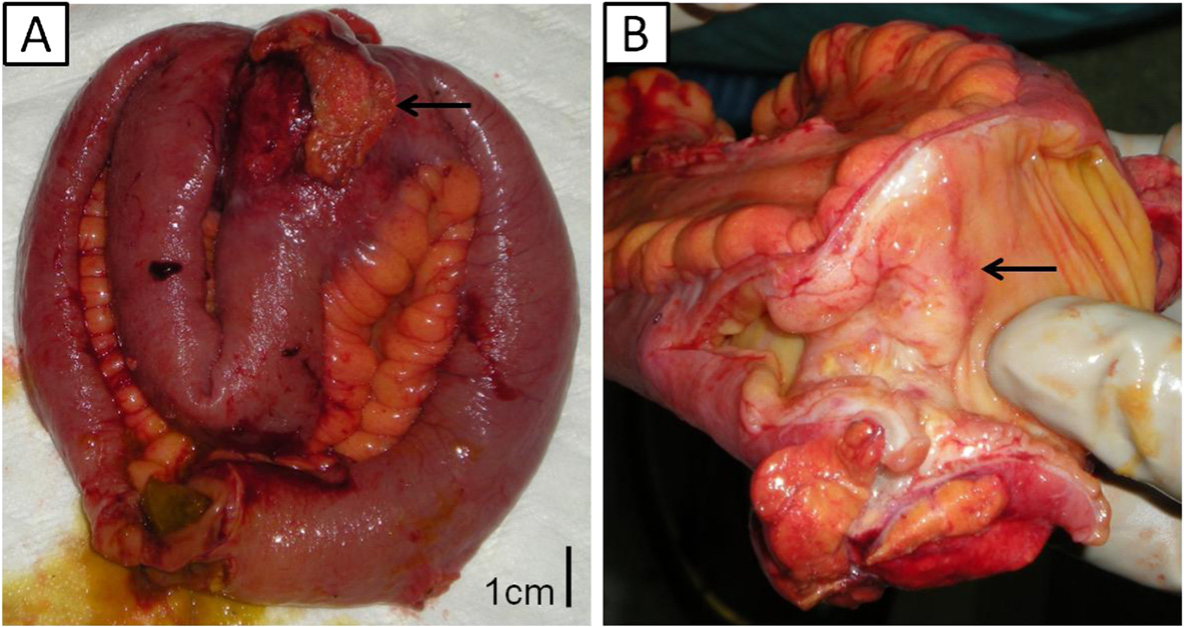
**Macroscopic views of the resected small bowel, where the tumor is noted (arrow).** (**A**) Tumor causing adhesion of the adjacent small bowel loop. (**B**) Intraluminal view, where the tumor causes circumferential-eccentric wall thickening of nodular formation, and thus concentric luminal narrowing.

The patient underwent emergency midline laparotomy and a circumferential-eccentric wall thickening of nodular formation was recognized at the level of the jejunum in the small intestine, causing concentric narrowing of the lumen. At the point of obstruction, adhered loops of small intestine were found (Figure 1). Partial excision of the upper ileum with side-to-side jejuno-ileal anastomosis was performed.

**Figure 2:**
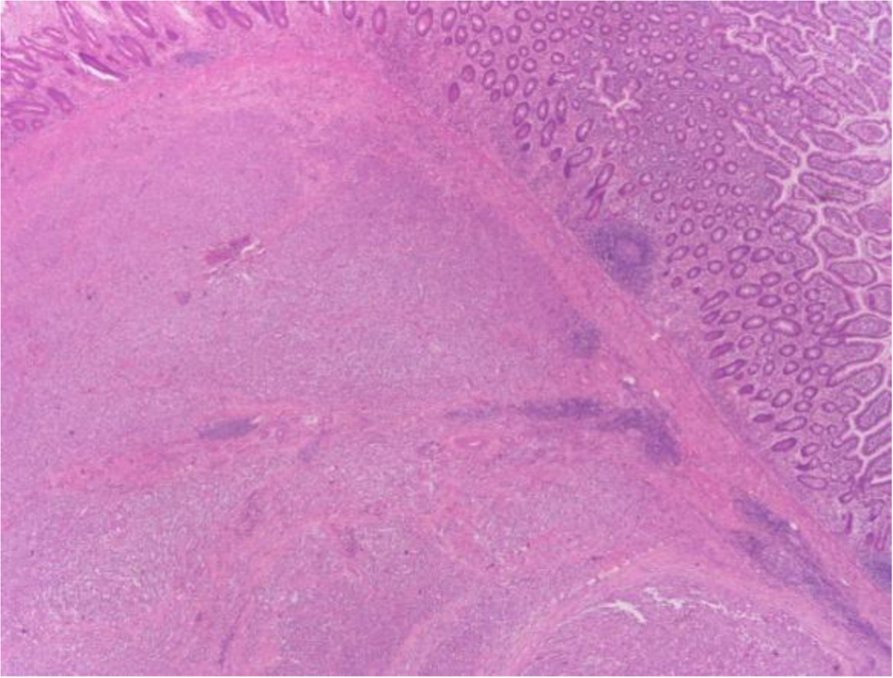
Tumor infiltrates the small bowel (hematoxylin-eosin staining × 40).

**Figure 3:**
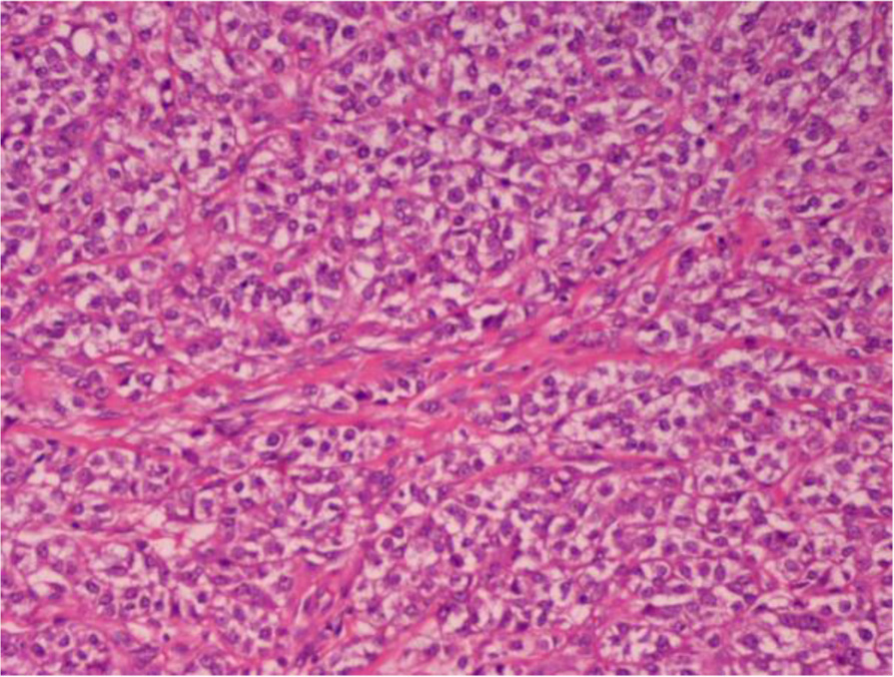
**Clear cell sarcoma.** Nests of epithelioid cells with eosinophilic or clear cytoplasm (hematoxylin-eosin staining × 200).

**Figure 4:**
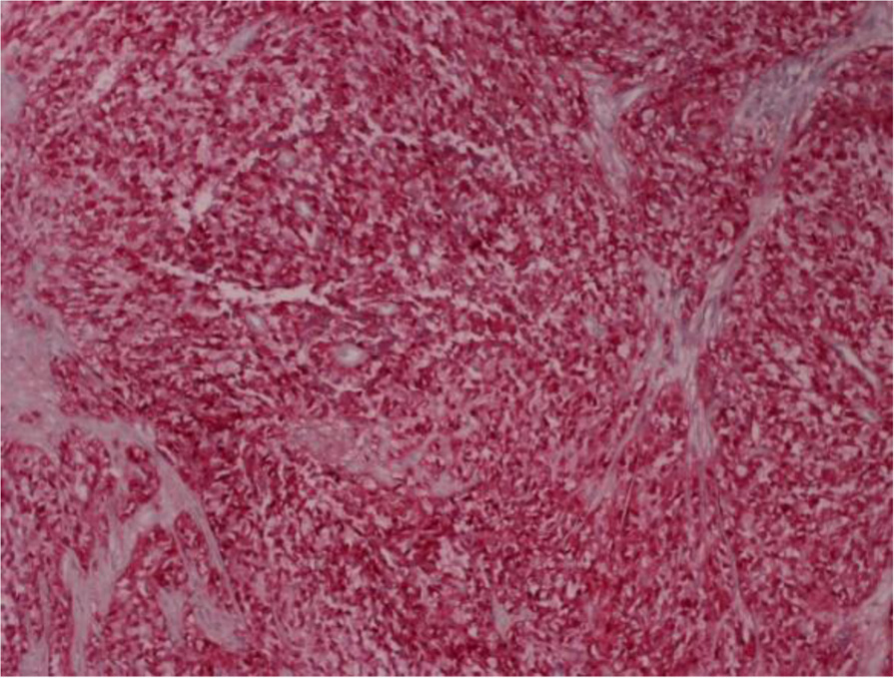
Neoplasmatic cells with positive expression of S-100 protein (S-100 staining × 100).

Pathological examination of the resected small bowel specimen revealed a 3 × 2 × 3 cm tumor. The microscopic examination showed an epithelioid neoplasm which was diffusely infiltrating the bowel wall in sheets and nests. The tumor cells had eosinophilic or clear cytoplasm and vesicular nuclei. In some areas, nuclei with nucleoli were observed. The mitotic rate was approximately eight mitoses per 10 high power fields (Figures 2 and 3). The immunohistochemical profile of the tumor was positive for S-100 protein (Figure 4), focally positive for epithelial membrane antigen (EMA) and weakly positive for synaptophysin at some locations and negative for pankeratins AE1/AE2, GFAP, HMB45 and MART-1. *EWRS1* gene rearrangement was observed during cytogenetic analysis and 12 out of 12 excised lymph nodes were free from neoplastic infiltration.

The patient had an uncomplicated post-operative course and left in good general condition after seven days of hospitalization. Twenty months post-operatively the patient remains asymptomatic and has no clinical manifestation of the disease.

## Discussion

Clear cell sarcoma is a rare type of malignancy, that shows features of melanocytic differentiation and most commonly affects tendons and aponeuroses of the distal extremities, particularly the foot and ankle (43%) and the knees, wrists and hands. Typically it presents as a slow growing tumor. In rare occasions it primarily affects the gastrointestinal tract [5]. A total of 21 GI CCS cases have been reported in the literature, more often arising in the ileum (10 cases) and less commonly in other sites, such as the jejunum (six cases) the duodenum (one case), the stomach (one case) and the colon (three cases). The present one is the seventh well-documented case of CCS arising in the jejunum reported in the literature. The median size of the reported cases is 5 cm and the average age of the patients at diagnosis is 39 years old [6]. A slight female predominance has been observed (male over female ratio is about 0.8). These characteristics are similar to the CCS of tendons and aponeuroses as presented in one of the largest and latest series [7].

The presentation of disease consists of intestinal obstruction, anemia or gastrointestinal bleeding [8]. Usually the patient is managed at an emergency setting due to intestinal obstruction, making surgical excision of the tumor the first step in its diagnosis and treatment. At laparotomy, GI CCS tumors typically appear as ulcerated masses involving the intestinal wall layers in full thickness. In some cases, though, the gross appearance is that of a polypoid lesion.

The pathological diagnosis of CCS in the gastrointestinal tract is often difficult, due to the inconsistent expression of melanocytic markers and its unusual site of presentation [6], requiring more than one approach. The common histologic features are similar to CCS affecting the tendons and aponeuroses. Usually the tumor consists of fascicles of small rounded cells which are separated by fibrous septa. They have abundant clear or eosinophilic cytoplasm and vesicular nuclei with prominent nucleoli. However, the majority of cases of CCS of the soft parts are characterized by expression of melanocytic markers (HMB45, A103, MITF and so on) more often than gastrointestinal CCS, where most tumors are negative for these markers [4]. In addition, a histologic variant of gastrointestinal CCS has been described, where the presence of scattered osteoclast-like multinucleated giant cells is the dominant feature [9]. Thus, there is not a general agreement on the histological profile of GI CCS. However, there are immunohistochemical characteristics that may provide clues to the differential diagnosis of the tumor. Strong S-100 protein positivity points to the exclusion of a gastrointestinal stromal tumor (GIST) [10], which accounts for most of the mesenchymal tumors at this site. In addition, the presence of EWS-ATF1 fusion transcript [3], resulting from a chromosomal translocation t(12;22)(q13;q12), when demonstrated in cytogenetic analysis is the only reliable marker for differentiating CCS from malignant melanoma, since most morphological, immunohistochemical and ultrastructural features are similar.

In CCS of tendons and aponeuroses, the commonly accepted management is wide surgical resection of the tumor with or without adjuvant radiotherapy. Metastatic tumors exhibit almost no response to systemic chemotherapy, as shown in a retrospective study of 24 cases [11]. However, there is no consensus on the management of GI CCS and most of the details about the management of these patients are usually not reported. Our case was managed based on the National Comprehensive Cancer Network (NCCN) practice guidelines for soft tissue sarcomas of the GI tract. The patient did not receive any chemotherapy or radiotherapy and was scheduled for follow-up visits (including abdominal, thoracic CT scanning) every three to six months.

Overall, GI CCS has an aggressive behavior, with a high incidence of local (lymph node) and distal (most commonly liver) metastasis. In 55% of the cases reported, lymph node metastasis was present at the time of diagnosis and only one case had metastasis to the liver along with regional lymph node metastasis. During the follow-up, liver metastasis developed in 32% of the patients reported, 27% have died and only three patients (14%) showed no evidence of the disease after the resection of the primary lesion. However, the detailed follow-up is not reported in many cases, which possibly leads to underestimation of the morbidity and mortality of the disease. Overall, the behavior is consistent with CCS of tendons and aponeuroses [12], although the latter metastasizes more commonly to the lungs or bones, a difference probably resulting from the different vascular drainage of the two different primary sites. In this case, however, all of the 12 lymph nodes resected were disease free and at the 20-month postoperative follow-up, still showed no recurrence of the disease. Unfortunately the small number of cases with adequate follow-up does not allow the determination of specific prognostic factors.

## Conclusion

Clear cell sarcoma of the gastrointestinal tract is a rare neoplasm with poor prognosis. The diagnosis is based on specific histological, immunohistochemical and genet characteristics. The mainstream treatment is surgical resection of the tumor, while there is no evidence about the effectiveness of adjuvant chemotherapy and radiotherapy.

## Consent

Written informed consent was obtained from the patient for publication of this case report and accompanying images. A copy of the written consent is available for review by the Editor-in-Chief of this journal.

## Abbreviations

CCS: Clear cell sarcoma;CT: Computerized tomography;EMA: epithelial membrane antigen;GI: Gastrointestinal tract;GIST: gastrointestinal stromal tumor;NCCN: National Comprehensive Cancer Network

## Competing interests

The authors declare that they have no competing interests.

## Authors’ contributions

KL took part in the care of the patient, manuscript composition and submission. AP and VL collected data, performed the literature research and manuscript composition. MT carried out the pathologic examination and performed the histologic studies. GC took part in the care of the patients, and the coordination and final editing of the manuscript. All authors read and approved the final manuscript.
